# Using Stakeholder Perceptions to Inform Future Efforts to Implement Mental Health First Aid Training in China: A Qualitative Study

**DOI:** 10.3389/fpsyt.2021.557282

**Published:** 2021-04-15

**Authors:** Shurong Lu, Yanling He, Kendall Searle, Pilvikki Absetz, Brian Oldenburg, Nicola Reavley

**Affiliations:** ^1^Jiangsu Provincial Centre for Disease Control and Prevention, Nanjing, China; ^2^Nossal Institute for Global Health, Melbourne School of Population and Global Health, University of Melbourne, Melbourne, VIC, Australia; ^3^Shanghai Mental Health Centre, Shanghai, China; ^4^Centre for Mental Health, Melbourne School of Population and Global Health, University of Melbourne, Melbourne, VIC, Australia; ^5^Institute of Public Health and Clinical Nutrition, University of Eastern Finland, Kuopio, Finland; ^6^Collaborative Care Systems Finland, Helsinki, Finland

**Keywords:** implementation, scale-up, mental health, Mental Health First Aid, evidence-based intervention, qualitative research

## Abstract

**Background:** The Mental Health First Aid (MHFA) training program has been widely implemented in many high-income countries. Evidence on the adaptation of this and other similar programs in resource-constrained settings like China is very limited. This study aimed to explore the views of key stakeholders on the implementation issues and contextual factors relevant to the scale-up of MHFA in China.

**Methods:** Informed by the *Consolidated Framework for Implementation Research*, five implementation domains of intervention characteristics, characteristics of individuals, contextual adaptation, outer and inner setting, and implementation process were investigated through semi-structured in-depth interviews. Twenty-four stakeholders with diverse expertise in the Chinese mental health system were interviewed. Transcripts were coded using NVivo 12 software and thematically analyzed.

**Results:** Fifteen themes and 52 sub-themes were identified in relation to the five domains. Participants saw MHFA as meeting the need for more evidence-based interventions to improve population mental health. Previous participants in MHFA training were satisfied with the course, but their intentions to help and levels of self-efficacy varied. Contextual adaptation of course content, delivery formats, and financing models, was seen as essential. External health policies and some socioeconomic factors (e.g., improved living conditions) were perceived as potential enablers of scalability. Low levels of engagement in health interventions and lack of supportive social norms were identified as potential barriers while executive support, quality control, and sustainable funding were viewed as facilitators of implementation.

**Conclusion:** MHFA training meets some very important current societal and public health needs in China. To achieve its potential impact, significant contextual adaptation is required, particularly in terms of course content, delivery formats, and financing models. Overcoming low levels of engagement in community-based mental health interventions and combating stigma will also be critical for its scale-up.

## Introduction

Mental, neurological, and substance use disorders affect a significant portion of the global population with a high burden, particularly in low- and middle-income countries (LMICs) (1). The latest epidemiological data show increases in the prevalence of these disorders in China, with an estimated 12-month prevalence of any of the above disorders of 9.3% in 2013 compared with 1.1% in 1982 (2), and a projected increase of 10% in the disease burden between 2013 and 2025 (3). In both developed and less-developed countries, the number of people with untreated mental disorders far outweighs those that receive treatment. In China, this unmet need constitutes an ongoing challenge to the mental health system (4).

Many factors, both individual and structural, interact to influence people's mental health service use (5). Among these factors, mental health literacy—defined as “knowledge and beliefs about mental disorders which aid their recognition, management or prevention” (6)—has been associated with improved attitudes and intended helping behaviors towards people with mental illness (7). Higher mental health literacy has been found to be a predictor of mental health service use (8). Unfortunately, evidence shows that Chinese people, including both laypeople and general health professionals, often have poor mental health literacy (9, 10) and high levels of stigma remain a significant problem (5, 11).

In recent decades in high-income countries (HICs), concerns about the contribution of poor mental health literacy and stigma to the mental health treatment gap have led to the development of interventions designed to address these issues (12). These interventions include the Mental Health First Aid (MHFA) training program, which focuses on training members of the public to provide mental health first aid (i.e., the help offered to a person with a mental health problem or crisis until appropriate professional help is received or the crisis resolves) (13). The content of the MHFA training course is based on a series of guidelines developed using the Delphi expert consensus method (14). In an MHFA course, people who are qualified to independently deliver courses are called *MHFA Instructors* (Instructors), and people who have completed an MHFA course are called *Mental Health First Aiders* (MHFAiders).

Since its inception in 2000 in Australia, MHFA has evolved into a global movement and is now implemented in over 27 (mostly English-speaking high-income) countries (14). In most countries, local MHFA organizations use a “*train the Instructor model*” that Instructors pay for their Instructor training and then charge their MHFAider trainees or are funded by their organizations to run training. This model (see Supplementary File 1 for details) has been found to facilitate the dissemination of MHFA in these countries.

MHFA programs have been extensively evaluated and shown to improve knowledge, mental health first aid intentions and confidence and reduce stigma (15, 16). Several small studies conducted in Chinese-speaking communities in Hong Kong and Australia have shown similar effects (17, 18). However, evidence in LMICs on how best to sustainably implement MHFA is still limited (13), although such countries are likely to benefit from evidence-based interventions developed in HICs with a greater budget allocated to health care and preventative services (19).

The Shanghai Mental Health Center, a specialized mental health institution in China, started to conduct the *Standard MHFA Training Course* in mainland China in 2017 after authorization by the *Mental Health Association of Hong Kong*. As of December 2020, there are 13 trainers in Shanghai and 30 training sessions have been delivered to 759 participants. The latter adopted the MHFA program from Australia and drew up the Chinese curriculum in 2004, maintaining much of the original format (14). Hong Kong and Australia have largely similar community-based mental health systems (20, 21); however, the mental health service system in mainland China is still largely hospital-centered (21, 22). Cultural understanding of mental health may also vary between Hong Kong and mainland China (e.g., In China, it may be more common to relate mental wellbeing to a harmonious relationship with others in the social context, rather than to an individual's growth and autonomy) (23).

This paper reports on a qualitative study that was undertaken to understand the implementation issues and contextual factors among key stakeholders in order to promote future scale-up of MHFA in China in a culturally appropriate way.

## Methods

### Study Setting

Semi-structured in-depth interviews were conducted in 2019 in Shanghai, China. As one of the most developed metropolitan regions of China, Shanghai has a relatively well-resourced mental health system and the ability to implement high-quality interventions (22). Due to its long history of openness and internationalization, Shanghai may also be more likely to be a pioneer adopter of an international intervention such as MHFA. Shanghai residents may also have higher than average levels of mental health literacy and greater interest in interventions in this field (24).

### Participants and Recruitment

Participants were potential key stakeholders for the wider implementation of MHFA in China. We employed maximum variation (in terms of gender, age, occupation, geographical region) and purposive and snowball sampling strategies in order to increase the likelihood that the findings reflect a wide range of views and perspectives. Participants were recruited from diverse mental health service settings *via* personal contacts or through MHFA training sessions happening in Shanghai during the study period. Participants were encouraged to introduce other eligible individuals they knew. Recruitment of participants was discontinued until data saturation was reached, i.e., when no further new information was obtained in subsequent interviews.

Twenty-four participants (women, 50%) were interviewed, representing the following stakeholders: psychiatrists (*n* = 10), mental health researchers (*n* = 3), mental health policy makers (*n* = 2), community mental health workers (*n* = 2), psychological counselors in universities (*n* = 3), human resource (HR) staff in large-scale enterprises (*n* = 2), and psychotherapist and social worker (*n* = 1, respectively).

**Figure 1:**
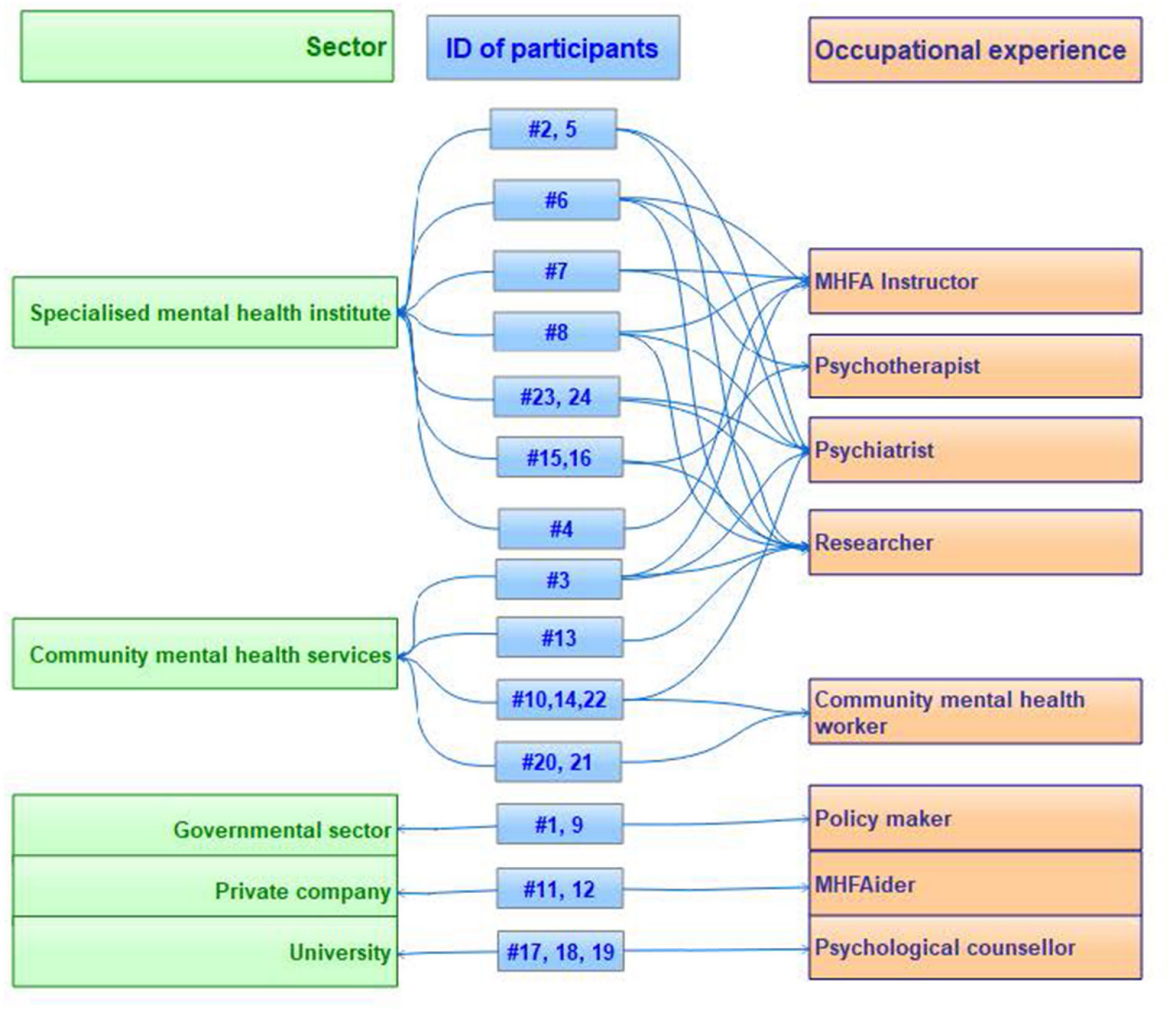
Background and occupational experience of participants (*n* = 24).

Figure 1 demonstrates the diversity of relevant settings and occupations of participants in this study. More than half of the participants (*n* = 13, 54%) had multiple occupational roles. Notably, 3 out of the 10 psychiatrists and the psychotherapist and social worker were also Instructors, and the two HR staff were MHFAiders. Seven participants had personal experience of MHFA training and were asked additional questions about their perceived satisfaction with their participation experience, motivations to participate, and self-efficacy as an Instructor/MHFAider.

The demographic and occupational characteristics of participants are shown in Table 1. Participants came from five provincial regions of China, aged between 28 and 53 years (mean 38.1, median 44), and all of them had a university education. On average, participants had 11.4 years (SD 7.5, range 3–27) experience in their relevant occupations.

**Table 1:** Characteristics of participants by occupation.

**Occupation**	**Region**	***N***	**Gender**	**Mean age in years (SD)**	**Average occupational years (SD)**
			**(%, women)**		
Psychiatrists^*^	Shanghai	6	50%	43 0 (6 1)	19 4 (7 3)
	Beijing	2			
	Heilongjiang	1			
	Henan	1			
Mental health researchers	Shanghai	2	67%	29 7 (2 1)	4 7 (0 6)
	Chongqing	1			
Mental health policymakers	Shanghai	1	0%	39 5 (0 7)	5 0 (5 7)
	Beijing	1			
Community mental health workers	Shanghai	2	100%	43 0 (14 1)	4 0 (1 4)
Psychological counselors	Shanghai	3	67%	35 3 (3 1)	8 3 (3 5)
Human resource staff^#^	Shanghai	2	0%	35 5 (3 5)	14 0 (2 8)
Psychotherapist^*^	Shanghai	1	100%	40 0 (NA^£^)	11 0 (NA^£^)
Social worker^*^	Shanghai	1	100%	31 0 (NA^£^)	5 0 (NA^£^)
Total		24	50%	38 1 (6 8)	11 4 (7 5)

* *Three out of the 10 psychiatrists and the psychotherapist and social worker were also qualified Instructors*.

# *The two human resource staff were also MHFAiders*.

£ *Not Applicable*.

### Data Collection

Informed by the *Consolidated Framework for Implementation Research* (CFIR) (25), five key implementation domains were identified, i.e., intervention characteristics, characteristics of individuals, contextual adaptation, outer and inner setting, and the implementation process. Given that MHFA is still at an early stage and has not been widely implemented in mainland China, the CFIR domains identified as being most relevant for the future scale-up of MHFA in China were selected, rather than those that are relevant during or after the implementation. These domains, their definitions, and relevant constructs of CFIR are presented in Table 2.

**Table 2:** Selected implementation domains, definitions, and constructs of the Consolidated Framework for Implementation Research (CFIR) used in the study.

**Domains**	**Definition of domains**	**Relevant constructs of CFIR**
1. Intervention characteristics	Stakeholders' perceptions of MHFA as an intervention, including its potential impact, advantages, and disadvantages over current practice	Relative advantage Evidence strength and quality
2. Characteristics of individuals	Motivations to participate, experience and satisfaction, and self-efficacy among people with participation experience of MHFA training	Knowledge and beliefs about the intervention Self-efficacy
3. Contextual adaptation	Components of MHFA that perceived to be adapted, tailored, or refined to meet the local needs of China	Adaptability
4. Outer and inner setting	Outer and inner setting-related factors that may facilitate the future implementation and scale-up of MHFA in China	External policies and incentives Peer pressure Implementation climate
5. Implementation process	Contextual factors that could support or hinder the implementation process	Planning Engaging Evaluating

An interview guide consisting of open-ended questions derived from the above implementation domains was developed for the interviews. These questions were organized in a flexible schedule, allowing for probing of further information and clarification where appropriate (see Supplementary File 2 for the full interview guide in English and Mandarin languages). Interviews were conducted in Mandarin by SL and audio-recorded with participant consent via face-to-face (63%, *n* = 15), over the phone (8%, *n* = 2), or WeChat (29%, *n* = 7)—a commonly used mobile application for social interaction in China. Interviews took place in a private room at the workplace of either the interviewer or participant and lasted 37–86 min (mean 53.1, SD 9.9).

Given that MHFA has been mostly implemented in HICs and the majority of people in China have limited knowledge of it at this stage, a document consisting of an introduction to MHFA and its implementation models in Australia and other HICs was sent to participants before their interviews (see Supplementary File 1 for the full text of this document in English and Mandarin languages). The research was approved by the Human Research Ethics Committee at the University of Melbourne (Ethics ID: 1853289.1) and the Ethics Committee at the Shanghai Mental Health Center (No: 2018-62).

### Data Analysis

Recorded interviews were transcribed verbatim and checked for accuracy by SL. Identifying data were removed from the transcripts prior to analysis, and participants were relabeled by their occupational roles and a number (e.g., Psychiatrist #1). Data were systematically managed, organized, and coded using NVivo 12 software. Data were analyzed using the thematic analysis method (26). SL developed and applied an initial coding framework. A discussion of this preliminary coding among the authors led to the iterative development of the final analytical framework. KS independently performed coding on 10% of transcripts. Fifteen themes and 52 sub-themes were identified, which were structured around the five implementation domains as listed in Table 3.

**Table 3:** Domains, themes, and sub-themes of implementing MHFA in China.

**Domains**	**Themes**	**Sub-themes**
1. Intervention characteristics	1.1 Perceived impacts	1.1.1 To Improve mental health literacy of people
		1.1.2 To enhance the capability of general health professionals
		1.1.3 To facilitate families of patients to provide better care
		1.1.4 To identify individuals with mental health problems
		1.1.5 To promote early detection
		1.1.6 Limited potential benefits
	1.2 Relative advantages	1.2.1 Systematically designed contents
		1.2.2 Inclusion of MHFA skills
		1.2.3 Standardized training procedure
		1.2.4 Active interactions in the course
	1.3 Relative disadvantages	1.3.1 No clear target in population or mental health problems
		1.3.2 Course content too complex
		1.3.3 Limited flexibility for Instructors
		1.3.4 Failure to consider mental health-related stigma
		1.3.5 Lacked localized contents
2. Characteristics of individuals (only participants with personal experience of MHFA training involved)	2.1 Experience and satisfaction	2.1.1 Instructors: all have delivered at least one course
		2.1.2 MHFAiders: just completed a standard course
		2.1.3 All satisfied with their experience
	2.2 Motivations to participate	2.2.1 Instructors: helpful for career development
		2.2.2 MHFAiders: nominated to do so by their employers
	2.3 Self-efficacy	2.3.1 Instructor: confident; tight for time; could be better
		2.3.2 MHFAiders: better knowledge but not confident to offer help
3. Contextual adaptation	3.1 Course contents	3.1.1 More flexibility
		3.1.2 Extra content on anti-stigma
		3.1.3 More cases from Mainland and community
		3.1.4 Enhancing skills development
		3.1.5 Optimizing the current course
	3.2 Course delivery	3.2.1 Involving new media and Internet
		3.2.2 Concerns about the effectiveness of these new methods
	3.3 Financing models	3.3.1 Limited affordability
		3.3.2 Charging may impede participation
		3.3.3 Possible alternative financing sources
4. Outer and inner setting	4.1 Policies	4.1.1 Most mental health policies are supportive
	4.2 Socioeconomic enablers	4.2.1 Attitudes change in a favorable way
		4.2.2 Increased knowledge and interests
		4.2.3 Improved living conditions
		4.2.4 The establishment of mental health network
	4.3 Pressure from existing programs	4.3.1 Few programs similar to MHFA exist
		4.3.2 Current public services already cover the content of MHFA training
5. Implementation process	5.1 Target population	5.1.1 Target institutions or organizations
		5.1.2 Instructor candidates need to have a medical background
		5.1.3 Any interested adults can be MHFAiders
	5.2 Barriers	5.2.1 Poor mental health literacy
		5.2.2 Low engagement in health education programs
		5.2.3 Lack of supportive social norms and values
		5.2.4 Shortage of mental health resources
	5.3 Facilitators	5.3.1 Executive support from the government and involved organizations
		5.3.2 Continued quality monitoring
		5.3.3 Development of a local implementation network
		5.3.4 Sustainable funding
		5.3.5 Offering a certificate
		5.3.6 other strategies for scale-up and sustainability

## Results

### Domain 1. Intervention Characteristics

#### Perceived Impacts and Challenges

Participants acknowledged that MHFA meets the enormous needs for mental health interventions in China and has the potential to improve the mental health of the population. Specifically, participants thought that, if successfully implemented, MHFA may have the following impacts: (1) to help to improve levels of mental health literacy among members of the public, which will improve the supportive social environment for people with mental illness; (2) to enhance the capability of health professionals in general hospitals and communities for prevention, detection, and treatment of mental disorders; (3) to support families of patients with mental illness in providing better care; (4) to help members of the public to identify and support individuals with mental health problems in specific settings, such as universities or workplaces.

Several participants worried that MHFA could be difficult to be put into practice in China and doubted that the implementation of MHFA would bring any impacts. The major perceived constraint was the upfront cost of training that would be borne by Instructors and MHFAiders. Another reason was limited understanding of the concept of mental health first aid in China. They also thought that it would be difficult for the program to realize its objectives without sufficient government support, which is currently the case with MHFA.

People attend a training course aiming to help others, and you are trying to charge them personally for this! Who is willing to do such a thing?!… I think this program is unlikely to succeed in practice. (Psychiatrist #2)

#### Relative Advantages and Disadvantages

Participants thought that, compared to other mental health programs in China, the most prominent advantage of MHFA was that it was systematically designed and could be applied to a wide range of mental health problems and diverse groups of people. Interviewed Instructors and MHFAiders also mentioned that the inclusion of opportunities to practice skills (as well as information provision) was attractive. Another advantage was the standardized training procedure, which was thought to be helpful to guarantee the course quality. Active interactions during the course were also positively appraised by previous Instructors and MHFAiders.

We lack such systematically designed interventions like MHFA, with both knowledge and skill practice included. It can be much more powerful for acquiring knowledge through systematic learning than by sporadic learning. (Psychotherapist #1/MHFA Instructor #4)

However, some of the above advantages were considered by other participants to be disadvantages. For instance, MHFA coverage of a wide range of mental health problems and diverse groups of people, one participant commented that “*targeting on all means no target at all*” (Psychiatrist #5). Several participants thought that the content included in the MHFA curriculum was too much and too complex for members of the public to master through a 12-h course (the length of a *Standard MHFA Course*).

Under a first aid circumstance, people usually depend on their instinctive reactions on the spot, with no time to think at all, so the simpler the better… Are you sure the public can remember so many points when needed? (Mental health researcher #1)

Several Instructors pointed out that the standardized training procedure used in MHFA gives them limited flexibility in course delivery and limits the performance of Instructors, who have different expertise and teaching styles. At least two participants mentioned that “*the current MHFA curriculum failed to consider the widespread stigma and discrimination toward mental illness in the Chinese context, but subjectively presumed that the general public is willing to offer help (to people with mental health problems), which may not be the case in reality*” (Community mental health worker/MHFA instructor #1). Instructors and MHFAiders consistently pointed to a lack of localized content in the current curriculum.

### Domain 2. Characteristics of Individuals

Interviewed Instructors had delivered at least one course in the previous year of the study and the two MHFAiders had just completed their course. Overall, they were satisfied with their participating experience, appraising it as attractive, interesting, and lively. Most Instructors indicated that they were motivated to be an Instructor because they viewed it as helpful for career development. The two MHFAiders participated because they were nominated by their organizations.

Instructors expressed high self-efficacy, though some of them felt that the course schedule was rather tight or that their course delivery could improve. MHFAiders thought the course would facilitate them to offer timely support to employees with mental health problems in their organizations, although they worried that their skills would not be good enough to help others, that they might make mistakes or might forget the material before getting a chance to offer help.

### Domain 3. Contextual Adaptation

#### Course Content

Several participants suggested more flexibility in course content, rather than adhering to the standardized format. For example, they advised dividing the current course into several packages by type of disorder or according to the needs of different groups of potential users (e.g., relapse identification-related content for families of patients with mental illness; early detection-related content for non-mental health professionals; and suicide or self-harm-related content for university students).

The training content should be determined by the actual needs of the audience. … Only in this way, a training course can be attractive to the audience and effective in practice. (Psychiatrist #5)

However, nearly the same number of participants took an opposite position on this issue as they thought that it was important for learners to have an overall understanding of common disorders, because it is hard to know what problems might happen to people around them.

Several participants suggested adding extra content on stigma, because, currently, many (Chinese) people lack adequate knowledge of mental health and believe myths about people with mental illness, including that they are unpredictable, dangerous, and immoral. A few Instructors and MHFAiders also advised including scenarios from Mainland China rather than from Hong Kong.

Several Instructors also provided suggestions on how to optimize the current curriculum, for example, to edit expressions and jargon from Cantonese style to Mandarin. The videos used in the current curriculum were seen as having a strong Hong Kong focus which they found distracting due to failure to reflect life in mainland China. Two Instructors further suggested adjusting the disorders included in the course (e.g., “*to include bipolar, but slightly reduce contents of substance abuse*,” MHFA Instructor #1). Several other participants addressed the need to culturally adapt the content in a general way.

The prevalence of mental disorders varies by countries and regions, so the focus of the course contents should also be different. (Psychiatrist #10/ MHFA Instructor #2)

People from the East and West are different. For example, Chinese people are more subtle, whilst foreigners are more open; our people ask for more privacy (when talking about mental health problems) but Westerners may feel okay to talk openly. … Thus, it is critical to culturally adapt the course content. (Psychiatrist #6)

#### Course Delivery

Although happy to attend the course, most Instructors and MHFAiders felt it was challenging for them to allocate 2 days to the training due to their full work schedule. Regarding this barrier, some participants suggested involving new media, for example, mobile apps for social networking, and the Internet to deliver the course. These formats may enable participation at people's convenience or by people from rural areas and remote regions (generally with limited mental health resources). These course delivery formats were thought to be appropriate for people who increasingly prefer to access resources and training *via* digital sources. Nevertheless, there were even more participants worrying about the effectiveness and quality of these new training formats.

Online courses are not good at interacting, their actual effectiveness is unknown either. For a new thing like MHFA, learning online might be not as effective as traditional face-to-face training. (HR staff member #1/ MHFAider #1)

#### Financing Models

As previously mentioned, in the implementation of MHFA in Western HICs, both Instructors and MHFAiders need to pay for their courses (unless the course is funded by a participant's organization). While a few participants thought that certain groups of people (e.g., families of patients with mental illness, people with strong personal interests or for career development) might be willing to pay for the course, most participants thought that many potential users would be less likely to participate if the financing model used in HICs was adopted in China, because they believed that while people are still struggling to live, they have limited interests in such learning.

It can be very difficult to implement a (health education) program if you charge participants, even in the most developed regions of China like Shanghai. (Mental health policy maker #1)

Alternatively, more than half of participants thought that government-funded services or institutions paying for staff to attend training were feasible options for financing.

### Domain 4. Outer and Inner Setting

#### Policies

Most participants acknowledged that the aims of MHFA align with the directions advocated in most current mental health policies in China, including the Mental Health Law, “Healthy China 2030 (i.e., a recent agenda of the central Government for health and development) and the China National Mental Health Working Plan (2015–2020).”

#### Socioeconomic Enablers

Several other socioeconomic factors were perceived as enablers, and the one that received the most recognition related to the societal change of attitudes toward mental illness. Overall, participants thought that members of the public were more accepting and less likely to avoid people with mental illness and more interested in mental health. They opted to attribute such changes to increased mental health knowledge and improved living conditions. Another social enabler was the mental health network in Chinese society, in which multiple government sectors beyond health were actively involved.

#### Pressure From Existing Programs

Most participants thought that, currently in mainland China, there were few programs similar to MHFA. However, several psychiatrists included in this study thought that the contents of MHFA had been fully covered by public mental health services in specialized psychiatric hospitals through activities like health education to patients with severe mental illness and their carers and regular campaigns on mental health knowledge dissemination among the general public.

### Domain 5. Implementation Process

#### Target Population

More than half of participants thought that Instructors should be mental health professionals, such as psychiatrists, psychotherapists, or psychiatric nurses or those with some medical background, in case misleading knowledge was transmitted. One interviewed psychiatrist explained that “*the large amount of improper health knowledge, which is being produced and disseminated almost every day, causes far more troubles than ignorance*” (Psychiatrist #5).

Almost all participants thought that any interested adult could be trained to be an MHFAider. Furthermore, participants thought that people who were staff in the health and education sectors, community practitioners, social workers, policemen, or volunteers were more likely to want to undertake training.

Though it was well-understood that the target population of MHFA in HICs are those interested individuals in the community (a wide concept, not limited to residential communities), more than half of participants thought that targeting organizations for recruitment would be more feasible, and such organizations could be general hospitals or community health centers. Given that MHFA was an “unfamiliar concept for most Chinese people, it was also seen as favorable to focus on universities and large-scale enterprises, as people in these were more likely to be open to innovation and better at learning new skills compared to other population groups.

#### Barriers

The most commonly mentioned factors that may impede the wide implementation of MHFA were stigma, discrimination, and prejudice, although most participants acknowledged the improvement in knowledge of and attitudes toward mental health in the past decades.

It is better (than decades ago), but members of the public are still apt to avoid or refuse people with mental health problems. (Community mental health worker #2)

Another major barrier was low engagement, which is common for most community health programs. Several participants emphasized that the engagement in mental health-related activities is usually even lower than in other activities, e.g., for chronic disease. As noted above, lack of time to attend training and the requirement to pay for attendance were also mentioned as reasons for low engagement in training.

The lack of supportive norms and values in Chinese society was highlighted. More than three participants mentioned that, unlike in HICs, at present, Chinese people have limited motivation to help others, not to mention that they need to pay for this. One researcher who was also a social worker mentioned that “*even if I would like to help, how can I?! Our culture advocates for keeping family scandals domestic, so it is difficult for outsiders (people beyond the family) to get involved*” (Social worker #1). The shortage of mental health resources, especially in those less developed and remote regions and rural areas in China, was perceived as another barrier.

#### Facilitators

Government support was thought to be an indispensable factor for the successful implementation of a program in China, because such support has the potential to encourage supportive policies and funding, and supported programs, particularly health-related ones, are usually seen as better quality. Similarly, executive support and alignment with the priorities of the organization where the program would be implemented were thought to be essential.

The importance of continuing to monitor course quality was addressed. One psychiatrist took the recent cancellation of a social training program for psychological counselors in China^1^ as an example to illustrate the possible consequences of poor quality control.

To achieve a wide implementation, the development of a local implementation network with relevant organizations was thought to be necessary. Local health sectors and specialized mental health hospitals, professional associations or groups, mental health-related social organizations, or traditional and new media were identified as potential members of such networks.

As mentioned in the contextual adaptation domain, most participants doubted the feasibility of the financing model of HICs being applied in China. The necessity of developing a sustainable financing model that may work for China was highlighted, but no specific suggestions were given.

Offering a completion certificate after an MHFA course was commonly practiced in HICs, but participants in this study had varied opinions on the value of this practice. Instructors, researchers, and psychological counselors were more supportive, while others were more hesitant as they thought the certificate would bring neither financial benefits nor career promotion. At least two participants expressed their concerns about possible improper utilization of the certificate, for example, the risk of it being seen as an approval to conduct psychological counseling.

Several participants also proposed that, in the long run, it was essential for MHFA to be integrated into the current mental health system. However, almost all participants thought that this would only be possible after the program has shown effectiveness. Several factors, including shortage of mental health professionals, extra burden on already heavy workloads, and limited executive support, were raised as obstacles to achieve this goal.

Several other strategies for scale-up were proposed, including (1) highlighting the potential benefits for self-help (besides helping others); (2) starting with certain settings like universities or workplaces in economically developed metropolitan areas; (3) advertising widely through various media in order to raise the awareness of this program; and (4) offering free courses to the public at the early stage.

## Discussion

Based on the views and perceptions of key stakeholders, this study investigated the implementation issues and contextual factors relevant to the future implementation of MHFA in China. The findings of this study can provide guidance on how best to adapt and implement MHFA in China in a culturally appropriate way. It also offers learnings for adapting population-based mental health interventions that have shown benefit in HICs to lower-resource settings with higher levels of stigma and reluctance to engage in mental health education interventions.

### Consistency and Divergency

Most participants agreed that the implementation of MHFA in China aligns with the societal and public health needs for more evidence-based mental health interventions, and it also aligns closely with recent mental health policies in the country (“*Healthy China 2030*,” for example). However, most interviewees agreed with the need to include locally appropriate course content and to consider how such a program could be funded and supported. These findings indicate directions that are likely to be used by program implementers to effectively adapt MHFA for the Chinese context.

Some participants favored simplified course content, media/Internet course delivery, and more flexibility for Instructors; however, others preferred a comprehensive approach, worried about the actual effectiveness of online training, and believed that limited flexibility for Instructors would enhance fidelity and produce better outcomes. Evidence suggests that stakeholders often have different values and perspectives on effective public health interventions (27). For example, practitioners often find evidence-based interventions difficult to conduct in community settings, especially when there is limited information about how to adapt programs to the local context, and health policy makers and program implementers are often reluctant to consider “new” interventions when effectiveness has not been demonstrated in their particular setting. Therefore, a better understanding of these divergent perceptions of stakeholders as well as the development of strategies to comprehensively address them should be taken into consideration in future implementation. Such dynamic interaction between the features of MHFA as a program, its potential users, and the local setting will facilitate the uptake of this program in a new cultural context (28).

### Adaptability vs. Fidelity

Adaptability and fidelity are central concerns of implementation research in public health, although their relative value has been controversial (29). Contextual adaptations are often necessary to improve stakeholder buy-in, increase the program's relevance for local populations, and facilitate the delivery of the intervention to the target population (30). Meanwhile, evidence shows that fidelity to the original intervention improves effectiveness, whereas significant modifications or deletions can diminish effectiveness (29).

This study identified several key adaptations of MHFA to be implemented in China, including those related to course content, delivery formats, and financing models. Some of these adaptations increase the likelihood of reaching a wider population or improving the acceptability of the program. A good example is to include extra anti-stigma components in the curriculum. In conceptualization, designers of MHFA presumed that people are willing to offer help to someone with mental illness if they possess relevant knowledge and skills. Originally, the anti-stigma content was not separately listed in the course, but embedded in the whole process of providing mental health first aid, such as to be empathetic, non-judgmental, and respectful to the person (14). Nevertheless, Chinese participants thought that, when combatting mental health-related stigma in China (11), a more direct approach may be necessary.

Evidence suggests that, in many LMICs, experiences of stigma, discrimination, and human rights abuses due to mental illness are common and severe (31). Multi-nation epidemiological studies have also shown higher rates of reported stigma among people with mental disorders in developing countries than in developed ones (32). Therefore, it seems necessary to include more anti-stigma content in the MHFA curriculum for China. Such adaptations may substantially enrich the content of MHFA to be implemented in LMICs, as well as further enhance its efficacy as an anti-stigma program.

While maintaining fidelity to achieve outcomes can result in research-based models' poor fit with the real world, improper adaptations may result in program drift away from the core elements needed to achieve designed outcomes (30). For example, some participants proposed allowing more flexibility to the systematically designed MHFA curriculum by splitting the courses into several packages targeting different disorders or people with particular interests. Such cultural tailoring might help to attract more attendance; nevertheless, it may blur the core elements of MHFA that make it distinguishable from other interventions and may also reduce its effectiveness.

### Other Health Workers as a Target Population

In addition to delivery in the community, some study participants suggested that health care settings, such as general hospitals or community health centers, could be suitable for MHFA implementation. If so, other health workers (i.e., those not directly involved in mental health) offer a potential first-line audience of MHFA in its wider implementation. This is very different from the practice in HICs, which generally excludes health workers (mental or other) (13). This may be partly explained by the differences in the mental health system between China and HICs. Western HICs such as Australia have relatively well-developed community mental health services (21), and most health practitioners in these countries have undertaken basic mental health training. In comparison, mental health services in China mostly remain hospital-based (22) and the recovery-oriented community mental health system is still in its early stages of development (33). Besides mental health specialists, most general health workers in China lack the skills and capability to recognize when a person is developing a mental illness in a timely manner or to provide needed support (10). However, these health workers have a higher chance of coming into contact with people with mental health problems than the general public, and frequently they act as gatekeepers for early detection of mental illness (34). Therefore, to have this group of people as the target population in the implementation process is of practical significance in enhancing early detection and narrowing treatment gap for mental disorders.

### The Role of MHFA in Behavior Change

Shonkoff stated that “the gap between what we know and don't do, is much larger than the gap between what we know and don't know” (35). By design, MHFA is a mental health intervention aiming to fill the “Know-Do” gap. It includes not only dissemination of mental health first aid knowledge but also promotion of behavior change (i.e., enabling people to offer help to someone in need and promote help-seeking) (13). However, this aspect of MHFA was not typically recognized across the study participants. Compared with the high endorsement among Instructors/MHFAiders, psychiatrists were more likely to understand MHFA as a very traditional health intervention, largely focused on raising awareness or providing knowledge. Accordingly, they perceived that such an approach would have quite limited benefits.

This finding suggests that behavioral change-oriented programs like MHFA are still relatively new among mental health stakeholders in China and their value is yet to be fully appreciated. A similar issue has also been reported in interventions for non-communicable diseases in China, which heavily rely on “what is known/said,” rather than on “what is done” (36), though research has repeatedly indicated that a change in knowledge or attitudes will not necessarily lead to a change in behavior (37). Therefore, during the wider implementation of MHFA, it may be useful to address the role of MHFA in promoting behavior change, i.e., the mental health first aid actions that are central to the program.

### Strengths and Limitations

This study is the first formal effort to consider how a program like MHFA could be culturally adapted and taken to scale in a resource-constrained setting like China. The study participants were all identified as having relevant knowledge about the future development and implementation of MHFA in China. Their diverse roles in relation to the field of mental health settings and service delivery provided a wide range of views and perspectives. Nonetheless, several limitations of the study should be considered. Firstly, participants were mostly from metropolitan regions of China (87.5%, Shanghai and Beijing), so the study findings are likely to be most relevant for urban areas compared to rural or less developed regions, although these regions are likely to be those with the highest demand for programs such as MHFA. Additionally, the study participants are unlikely to be representative of the full range of stakeholders who will influence future implementation. Although every effort (e.g., wording in a neutral way, taking a neutral position during interviews and data analysis) was taken to minimize possible information bias, the way that participants expressed their opinions might be positively biased due to their prior familiarity with the interviewer or the MHFA training program.

## Conclusion

As an evidence-based mental health intervention in developed countries, the MHFA program could meet some of the urgent societal and public health needs in China to improve mental health care delivery and outcomes in the population. However, to achieve this promise in a very different society and context from where MHFA was originally developed, significant contextual adaptation is required, particularly in terms of course content, delivery formats, and financing models. To enable this adaptation, it is very important to understand the barriers and facilitators to wider implementation, as well as how to address these in the Chinese context, in particular, stigma and a low level of engagement in mental health education interventions. As the re-development of MHFA currently underway and a randomized controlled trial funded and planned, further reflection on the findings of this study and other lessons from this research will contribute to the evidence base for cultural adaptation and implementation of health education interventions in China.

## Data Availability Statement

Raw qualitative data cannot be shared due to ethical restrictions. Anonymized quantitative data will be shared on a reasonable request to the corresponding author.

## Ethics Statement

The studies involving human participants were reviewed and approved by the Human Research Ethics Committee at the University of Melbourne (Ethics ID: 1853289.1) and the Ethics Committee at the Shanghai Mental Health Center (No. 2018-62). The participants provided their written informed consent to participate in this study.

## Author Contributions

SL designed the study, developed the interview guide, coordinated and conducted fieldwork, analyzed data, and drafted the manuscript. YH was the key liaison for the recruitment of participants. KS conducted double-coding on 10% of transcripts and checked key themes. NR and BO advised and supported the development of the study design. PA provided input to the final draft. All authors read and approved the final version of this manuscript for publication.

## Conflict of Interest

The authors declare that the research was conducted in the absence of any commercial or financial relationships that could be construed as a potential conflict of interest.

## Footnotes

^1^To satisfy the increasing demand of psychological counseling services, the Chinese Ministry of Labour (the currently Ministry of Human and Social Affairs) authorized qualified social organizations to carry out training programs of psychological counselor since 2001. However, these social training programs were canceled in Sep. 2017, partly due to their poor training quality.

## Supplementary Material

Supplementary File 1 Introductory document on MHFA in English and Mandarin languages.

Supplementary File 2 Full interview guide in English and Mandarin languages.
