# Supplementary material for: Using Stakeholder Perceptions to Inform Future Efforts to Implement Mental Health First Aid Training in China: A Qualitative Study
Source: Front Psychiatry. 2021 Apr 15;12:557282. doi: 10.3389/fpsyt.2021.557282 (PMC8081887; doi:10.3389/fpsyt.2021.557282)
Supplement: Supplementary File 1 — Introductory document on MHFA in English and Mandarin languages. [file Data_Sheet_1.docx]

Introduction to the Mental Health First Aid training program and its implementation models in high-income English-speaking countries

**Cover letter**

Dear experts,

The interview aims to explore how should the MHFA program be implemented in a culturally appropriate way under China's cultural background and existing health system.

Given that MHFA training is still at a very early stage in China, we wrote this introductory document for you to facilitate a better understanding of this program and to invoke insights for its sustainable implementation in China.

This is the outline of this document:

Section 1. Overall introduction

1.1 What is the MHFA training program?

1.2 The history of its development

1.3 Evidence on its effectiveness

Section 2. MHFA training courses

2.1 Audience

2.2 Contents

2.3 Courses

2.4 Course delivery

Section 3. The implementation models of MHFA in Australia and other HICs

Please read this document before the interview and feel free to ask if you have any questions, now or during the interview.

Thank you in advance for your interests and acceptance of this interview.

Looking forward to meeting you and hearing your opinion.

# Section 1 Introduction to the MHFA program

## 1.1 What is the MHFA training program?

The MHFA training program conducts courses which teach members of the public how to provide mental health first aid, which has been defined as “the help offered to a person developing a mental health problem, experiencing a worsening of an existing mental health problem or in a mental health crisis; the first aid is given until appropriate professional help is received or until the crisis resolves”.

## 1.2 The history of its development

The MHFA program was established in Australia in 2000 and has since spread rapidly in Australia and other countries around the world. MHFA has now expanded to every state and territory in Australia and 25 (mostly high-income English-speaking) countries and regions around the world.

MHFA courses teach mental health first aid strategies to members of the public in communities and workplaces using the Action Plan – ALGEE, which represents:

- **A**pproach the person, assess and assist with any crisis
- **L**isten and communicate non-judgmentally
- **G**ive support and information
- **E**ncourage the person to get appropriate help
- **E**ncourage other supports

It is important to note that the MHFA training is aimed at non-mental health professionals and the community, and the training course is about teaching people how to provide mental health first aid to others so that recipients can access professional mental health assistance promptly. Therefore, MHFA is not a therapeutic service and is different from peer support in the group.

## 1.3 Evidence on its effectiveness

Since it began in 2000, Mental Health First Aid Australia has been committed to evaluating its training programs using rigorous, scientific studies. A number of these evaluations were conducted by MHFA or by the Population Mental Health Group at the University of Melbourne. Evaluation studies have also been conducted by independent organisations.

These evaluations consistently show that MHFA training is associated with improved knowledge of mental illnesses and their treatments, knowledge of appropriate first aid strategies, and confidence in providing first aid to individuals with mental illness. Some studies have also shown improved mental health in those who attend the training, decreases in stigmatising attitudes and increases in the amount and type of support provided to others.

# Section 2 MHFA Training Course in English-speaking countries

## 2.1 Audiences of MHFA training

The MHFA courses can be broadly divided into two categories, depending on the training audience:

### 2.1.1 MHFA Instructors

The course aims to train people with relevant experience to become MHFA Instructors who will independently conduct MHFA course training at the community level in the future. Instructor applicants need to have some basic qualifications, including:

- Respect for people with mental health problems
- Enthusiastic of MHFA courses
- Excellent teamwork and communication skills
- Understanding mental health services
- Better social skills

### 2.1.2 General recipients/MHFAiders

This course is aimed primarily at non-mental health professionals and the community, in other words, any non-mental health professional and the general public interested in MHFA.

## 2.2 Contents of MHFA training

The MHFA courses teach people simple, practical first aid skills for helping a family member, friend, co-worker or another person who is experiencing mental health problems. MHFA courses will teach audiences how to listen and respond to someone with a mental health problem, even if they are experiencing a crisis. Audiences will learn how to help someone to access the support they might need for the successful management of symptoms as part of their recovery journey. This could include self-help books or websites, accessing support through their GP, their school or place of work, online self-referral, support groups, and more.

Specifically, MHFA courses can provide members of the community with:

- Skills in how to recognise the signs and symptoms of mental health problems
- Knowledge of the possible risk factors for these mental health problems
- Awareness of the evidenced-based medical, psychological and alternative treatments available
- Skills in how to give appropriate initial help and support someone experiencing a mental health problem
- Skills in how to take appropriate action if a crisis arises involving suicidal behaviour, panic attack, stress reaction to trauma, overdose or threatening psychotic behaviour.

## 2.3 Available MHFA courses

As the first country to establish the MHFA program, Australia has up-to-date MHFA training courses. Courses for MHFA Instructors are five days long and will be taught by two experienced MHFA Instructors from the organization named *MHFA Australia* (a not-for-profit organisation, whose role will be introduced in Section 3 of this document), and is based on face-to-face teaching. These courses for Instructors are of increased depth and breadth compared to courses for the general MHFAiders and are more demanding in skills development related to providing mental health first aid actions.

In comparison, courses for general MHFAiders are more flexible and diverse in form. For example, the number of current curriculum for general MHFAiders in Australia has grown to 10, including:

### 2.3.1 Core courses:

- Standard courses: 12-hour course for adults to assist other adults;
- Youth courses: 14-hour courses for adults to help young people;
- Seniors courses: 12-hour course for adults over 65 years of age;
- Youth courses: A classroom-based 3-hour course for young people to help their peers.

### 2.3.2 Courses on specific mental health issues

- Addressing suicide: a half-day course for adults to assist people who encounter suicidal thoughts and behaviour;
- Targeting non-suicidal self-injury: a half-day adult course to assist people with self-inflicted injury;
- For gambling problems: a half-day adult course to assist people experiencing gambling problems.

### 2.3.3 Skills updated courses

- For those who have already obtained the MHFA First Responder Certification, they can update their MHFA skills by completing a half-day workshop and receive another three years of continuous.

## 2.4 Training course delivery

In Australia, MHFA has developed several relatively flexible training formats to meet the needs of different groups of people.

### 2.4.1 Face-to-face

All of the MHFA courses mentioned above, including core courses, mental health-specific courses, and skills updates, can be conducted in face-to-face formats in all Australian states and territories (some courses may not be offered in individual remote areas).

### 2.4.2 eLearning + face-to-face

This form of training is aimed primarily at the standard 12-hour course for adults assisting other adults. Combined with a half-day face-to-face seminar, the e-learning part takes about 6-8 hours and can be divided into several sections. This "online learning and face-to-face" training method is suitable for the professional population, college students, and so on.

### 2.4.3 Online

This form of training is primarily for the overseas workplaces of Australian multinational organisations and institutes that have not conducted MHFA training in their home countries but are interested in providing MHFA training to members of the institute. These organizations or institutes can apply to *MHFA Australia* for an online MHFA training course.

# Section 3 The implementation models of MHFA in Australia and other HICs

Australia is the first country to establish and conduct MHFA training. *MHFA Australia*, a national non-profit organization that focuses on mental health training and research, is a major driver of the current development of MHFA globally. *MHFA Australia* operates under the management of the board of directors and is funded mainly by government or charitable funds, public donations, and its training fees.

*MHFA Australia* is responsible for the development and design of all MHFA training courses, including training manuals, and other relevant materials. This organization is also responsible for the recruitment and training of Instructors, who are going to carry out the recruitment and training of general MHFAiders in their communities or workplace scattered across Australia. Instructor participants are required to pay approximately RMB ￥10000-20000 for their training. The fee covers all training courses attended, a set of Instructor toolkit and ongoing support from the team of *MHFA Australia*. The instructor toolkit contains MHFA teaching materials that are necessary for MHFA training. The toolkit also includes supporting resources for mental health problems.

To maintain the certification of MHFA Instructors, all Instructors are required to:

- Regularly offer MHFA courses (at least three courses per year)
- MHFA manual for each participant
- Maintain the continued development of professional skills
- Payment of an annual membership fee of approximately RMB ￥500

Training courses for general MHFAiders are conducted independently by certified MHFA Instructors in their communities or organizations. Instructors can charge students for tuition fees, the amount of which varies depending on the location of the Instructor, the format of courses offered and the quality of the training. At the end of the course, participants can visit the website of *MHFA Australia* for a unified online assessment. People pass this assessment can obtain a certificate from *MHFA Australia*, which provides evidence for their knowledge and skills to act as an MHFAider. This certificate is valid for three years.

In Australia, the expansion of MHFA is based on a "decentralised Instructor training" model. This model is often used in health care and health education in high-income countries, and it facilitates the rapid and low-cost development of large numbers of people for projects. Compared to the model that all MHFA courses are centrally delivered and managed by one organization, the decentralised model brings stronger support from communities and organizations. In this model, Instructors firstly pay for the training, and then the participants they enroll or the organizations they serve to pay for the training they provide. Obviously, in this model, the quality of MHFA training depends largely on the ability and quality of the Instructors. Therefore, the selection of Instructors and quality control of the training they deliver are key factors affecting the effectiveness of MHFA.

*MHFA Australia* has entered into licensing and royalty agreements with organizations outside Australia that allow them to adapt their MHFA curriculum and implementation model to their specific contexts. Most English-speaking countries are currently running MHFA programs on a model similar to Australia's, as most of them are high-income countries with similar health systems and similar cultural backgrounds to Australia.

Despite its rapid development in recent two decades, MHFA raining has been mostly implemented in high-income English-speaking countries, and experience in low- and middle-income countries or non-English-speaking countries is still scarce. Given differences between China and these English-speaking countries in terms of health system, cultural background, language, and people's knowledge of and attitudes towards mental illness, it is important to tailor the MHFA training for Chinese contexts.

I look forward to hearing your opinions and insights in the coming interview.

Thank you very much for your support and participation!

精神健康急救项目

及其在高收入国家的实施模式介绍

尊敬的专家您好！

感谢您接受我们的访谈，探讨在中国文化背景和现有卫生体系的背景下，如何实施精神健康急救项目，并实现其可持续发展。

鉴于此项目在中国尚处于探索阶段，部分专家可能对其不是十分了解，所以我们将本文发送给您。通过本文，您可以简要了解精神健康急救项目及其在高收入国家的实施模式，以便您更好地了解该项目，并对其在中国的实施模式发表您的见解。

本文分共为三个部分：第一部分为项目概述；第二部分介绍项目培训相关内容;第三部分介绍该项目在澳大利亚及其他高收入国家的实施模式。

请您在访谈前阅读本文。您有任何问题，欢迎通过邮件或在访谈时进行询问。

# 第一部分 概述

MHFA是一项遍布全球的社区精神健康教育项目。参与者**通过参加精神健康急救课程学习，掌握**如何帮助那些精神健康正在出现问题、现有精神健康问题恶化或遭遇精神健康危机的人，直到他们获得适当的专业帮助或危机结束。

MHFA培训于2000年在澳大利亚创立。此后，在澳大利亚和和全球其他国家迅速传播。目前已经扩展到澳大利亚的每个州和领地，以及全球**25个国家和地区（多数为高收入的英语国家）**。

**ALGEE**是MHFA致力推广的**精神健康救助原则**的英文首字母缩写，分别代表：

- A (Approach the person, assess and assist with any crisis)：接近对方、评估风险和提供帮助；
- L (Listen non-judgmentally)：非批判性倾听；
- G (Give support and information)：提供支持和有用信息；
- E (Encourage the person to get appropriate professional help)：鼓励寻求专业帮助和治疗；
- E (Encourage other supports)：鼓励对方寻求其他方面的支持和帮助。

**需要注意的是，**MHFA的培训对象是非精神卫生专业人士和社区民众。培训内容是教授人们如何向他人提供精神健康紧急救助，以便受助者能够及时地获得专业的精神卫生帮助。因此，**MHFA不是一种治疗性的服务，也有别于小组同伴支持**。

对在高收入国家开展的MHFA培训的系统评估表明，MHFA课程培训可以有效提升大众对精神疾病的治疗、康复及其他相关精神卫生知识的认知水平，改善针对精神健康问题的不良态度，以及提升学员为精神疾病患者提供急救的能力和信心。研究结果还表明，参加培训的学员，其自身的精神健康得到改善，对精神疾病的负面态度减少，给他人提供的支持也有所增加。

# 第二部分 MHFA培训课程

## MHFA课程的培训对象

MHFA课程按照培训对象的不同，大致分为两类：

1. **针对MHFA培训讲师的课程**

课程目的是将有相关经验的人士培养成为日后能在社区层面独立开展MHFA课程培训的讲师。讲师申请人需要具备一些基本条件，例如：

- 具有对精神健康问题的人的尊重态度
- 热心提供MHFA课程
- 优秀的团队协作和沟通技巧
- 了解精神健康服务
- 较好的社交技能

1. **针对普通MHFA急救人员的课程**

这一类课程主要针对非精神卫生专业人士及社区人群。换言之，任何对MHFA感兴趣的18岁及以上的非精神卫生专业人士和普通民众均可参加。

## MHFA课程的培训内容

MHFA课程主要教授学员简单实用的精神健康急救技能，即如何在MHFA救助原则（此前提及的ALGEE原则）的指导下，帮助家庭成员、朋友、同事或其他正在经历精神健康问题的人，使其获得有效的管理疾病症状所需的支持，成为帮助他们的精神康复的一份子。

具体而言，MHFA培训内容包括：

- 识别精神健康问题的迹象和症状的技巧
- 精神健康问题的可能危险因素
- 基于证据的医学、精神和替代治疗方法
- 提供适当的初步帮助和支持经历精神健康问题的人的技能
- 出现涉及自杀行为、惊恐发作、对创伤的压力反应、过量服药或威胁精神病行为的危机情况时，采取适当行动的技巧

## 现有澳大利亚MHFA课程类型

在澳大利亚，针对MHFA培训讲师的课程为期5 – 5.5 天，由两名来自澳大利亚MHFA组织（这一组织将在MHFA实施模式部分予以介绍）、具有丰富经验的MHFA讲师进行授课，以面授的形式为主。与针对普通MHFA急救人员的课程相比，**针对讲师们的课程，在内容的深度和广度方面均有所增加，同时，在技能发展方面也要求更高**。

如前所述，针对普通MHFA急救人员的课程，与针对讲师的培训课程相比，在培训内容的深度和技能发展方面要低，但是，在形式上要更加灵活和多样。例如，目前在澳大利亚开展的针对普通MHFA急救人员的课程已发展至10种之多，包括：

1. **核心课程：**

- 标准课程：成年人协助其他成年人的12小时课程；
- 青年人课程：成年人为青年人提供帮助的14小时的课程；
- 老年人课程：成年人为65岁以上老年人提供帮助的12小时的课程；
- 青少年课程：青少年为他们的同龄人提供帮助的、基于课堂的3小时的课程。

1. **针对具体精神健康问题的课程**

- 针对自杀问题：为成年人提供半天课程，协助曾有自杀念头或行为的人；
- 针对非自杀性的自伤行为：成年人半天课程，协助有自伤行为的人；
- 针对赌博问题：成年人半天课程，协助遇到赌博问题的人；

1. **技能更新的课程**

MHFA急救人员认证的持证人员，可以通过完成为期半天的研讨会来更新其MHFA技能，并获得后续三年的持证资格。

## MHFA的培训方式

在澳大利亚，MHFA已经开发了几种相对灵活的培训方式，以满足不同人群的需要。具体而言，MHFA的培训方式包括：

1. **面授型**

上述提到的所有MHFA课程，包括核心课程、针对具体精神健康问题的课程和技能更新课程，在澳大利亚的各个州和领地均可通过面授的形式开展（个别偏远地区可能没有开展所有类型的课程）。

1. **“网上学习+面授”型**

这种培训形式主要针对成年人协助其他成年人的12小时的标准课程。网上学习部分需要大约6-8个小时（可以分成几个部分来完成），再加上为期半天的面对面研讨会。这种“网上学习+面授”的培训方式适用于职业人群、大专院校的学生等。

1. **在线培训型**

这种形式的培训目前主要针对澳大利亚跨国组织/集团在海外的工作场所，以及有兴趣为组织成员提供MHFA培训的组织或机构。这些组织或机构可以向澳大利亚MHFA组织申请MHFA在线培训课程。

# 第三部分 MHFA项目在高收入国家的实施模式

澳大利亚是全球最先开展MHFA培训工作的国家，其澳大利亚MHFA组织（MHFA Australia）也是目前全球MHFA发展的主要推动者。该组织是澳大利亚全国性的非营利性健康促进慈善机构，专注于精神健康培训和研究。澳大利亚MHFA组织在董事会的管理下运作，资金主要来源于政府或慈善基金、公众捐赠以及培训收费。

澳大利亚MHFA组织负责开发和设计所有MHFA培训课程，包括培训手册、课件及相关材料等。该组织还同时负责培训讲师的招募和培训。这些遍布澳大利亚境内不同的州和领地的培训讲师，负责各自所在地区或所在组织的普通 MHFA急救人员的招生和培训。讲师学员需要为此培训支付约1-2万元人民币。这笔费用涵盖了参加的所有培训课程、一套讲师工具包，以及澳大利亚MHFA团队的持续支持。讲师工具包包含MHFA教学材料，这些材料是其开展MHFA培训所必需的。工具包中还包括针对精神健康问题的一些支持性资源。

为了保持MHFA培训讲师的认证资格，讲师们在获得认证之后需要：

- 定期提供MHFA课程（每年至少开展三次课程）
- 为每位参与者提供MHFA手册
- 保持专业技能的持续提升
- 支付约500元人民币的年度会员费

针对普通MHFA急救人员的培训课程，则由获得认证资格的MHFA培训讲师，在其社区或组织内独立开展。讲师可以向学员收取学费，学费因讲师所在地区、提供的课程类型和培训质量等的差异而有所不同。课程结束后，学员可以登陆澳大利亚MHFA组织官网，接受统一的在线评估。评估合格者，即表示具备成为精神健康急救人员所需的知识和技能，可获得由澳大利亚MHFA组织颁发的认证资格证书。证书有效期为三年。

由以上介绍可见，**在澳大利亚，MHFA的推广是基于一种“培训讲师分散化”的模式**。这种模式在高收入国家中被普遍用于医疗保健和健康教育，有利于项目快速且低成本地培养大量人员。和所有MHFA课程都由一个组织来集中提供和管理的模式相比，这种模式能够带来更强大的来自社区或组织机构的支持。在此模式中，讲师首先付费接受讲师培训获得MHFA讲师资格，然后再收费培训普通学员或为组织机构提供服务。显然，**在此模式下，MHFA培训的质量主要取决于讲师们的能力和素质。因此，对于讲师学员的选择及其培训的质量控制，是影响MHFA效果的关键因素**。

澳大利亚MHFA组织与澳大利亚境外组织签订了许可和版税协议，允许这些组织根据具体情况对MHFA课程和实施模式进行调整。**目前，多数国家采用与澳大利亚类似的模式运行MHFA项目，因为它们多数为高收入国家，和澳大利亚的卫生体系和相近的文化背景相似。**

尽管MHFA近年来发展较快，但目前仍然主要集中在高收入英语国家。**在精神卫生资源相对匮乏的中低收入国家和非英语国家，实施经验仍然非常有限**。就中国而言，与高收入国家相比，我国无论是在卫生体系、文化背景、语言方面，还是在民众对于精神健康问题的知识了解程度、态度和求医行为方面，均有诸多差异。因此，**在中国开展和推广MHFA工作时，我们可以借鉴MHFA在高收入国家中的实施模式，但更需要充分考虑到我国的具体情况**。

期待听到您对于在中国实施和推广MHFA项目的精彩见解！
